# Carrageenan: structure, properties and applications with special emphasis on food science

**DOI:** 10.1039/d5ra03296b

**Published:** 2025-06-27

**Authors:** Faiza Jabeen, Zil-e-Aimen, Rimsha Ahmad, Sadullah Mir, Nasser S. Awwad, Hala A. Ibrahium

**Affiliations:** a Department of Chemistry, COMSATS University Islamabad Pakistan sadullahmir@comsats.edu.pk +92-03336781744; b Chemistry Department, King Khalid University P.O. Box 960, AlQura'a Abha Saudi Arabia; c Biology Department, King Khalid University P.O. Box 960, AlQura'a Abha Saudi Arabia

## Abstract

This comprehensive study examines the versatile applications of carrageenan, a sulphated polysaccharide derived from red seaweeds, across environmental remediation, biomedical sciences, and food technology. The study also explains carrageenan's structural characteristics, physicochemical properties and the three primary types: kappa, iota, and lambda. In wastewater treatment, carrageenan-based materials demonstrate efficacy in removing heavy metals, dyes, and organic compounds through adsorption and flocculation mechanisms. The biomedical potential of carrageenan is explored, highlighting its role in drug delivery systems, tissue engineering, and wound healing applications. The review also addresses carrageenan's significance in the food industry as a thickening agent, stabilizer, and preservative. While acknowledging potential drawbacks, including gastrointestinal concerns and environmental impacts of seaweed harvesting, the study presents a balanced analysis of carrageenan's applications. This review underscores carrageenan's potential as a versatile, eco-friendly solution across multiple sectors, contributing to advancements in sustainable technologies and biomedical innovations.

## Abstract 2

Carrageenan: structure, properties and applications.

## Graphical abstract

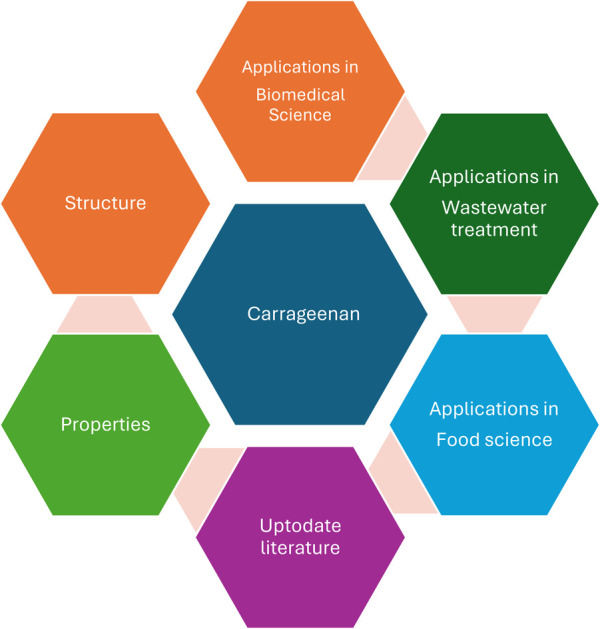

## 1. Introduction

Algae are photosynthetic organisms and belong to a complex and often debated classification system. They are primarily categorized into two types: (i) microalgae, which are small in size and located in littoral and benthic environments as well as all over the sea waters as phytoplankton, and (ii) macroalgae, commonly known as seaweeds, which are larger and typically inhabit the littoral zone.^1,2^ Seaweed belongs to multicellular organisms having a photosynthetic nature that can serve as a valuable biomass resource, potentially replacing terrestrial biomass in the production of biochemical products and biofuels.^3^ Algae are a great option for renewable resources because they grow quickly, have high photosynthetic productivity, can efficiently capture carbon dioxide, contain a lot of carbohydrates, and have low lignin content.^4–6^ Seaweed contains high levels of polysaccharides, storage polysaccharides, and mycopolysaccharides in its cell walls.^7^ Polysaccharides in algae are attracting attention because they are sustainable, available in large quantities, and have a unique chemical composition not found in other organisms.^8^ It encompasses a broad range of biopolymers obtained from various seaweeds, including ulvans (green seaweeds), alginates (brown seaweeds) and from red seaweeds. Due to their unique characteristics, such as gelling, thickening and other activities, including antiviral, anti-inflammatory, and anticoagulant effects, carrageenans and alginates are widely used in the pharmaceutical, food and cosmetics industries.^9^ Carrageenan, a sulfated polysaccharide derived from marine red algae, exhibits biodegradability, nontoxicity, and water solubility, making it a versatile material with applications in various fields.^10^ These polysaccharides consist of alternating units of d-galactose and 3,6-AG (3,6-anhydro-galactose) connected by α-1,3 and β-1,4 glycosidic bonds.^11–13^ Carrageenan is a sulfated polygalactan containing ester-sulfate (15–40%), making it an anionic polysaccharide. It is employed in the food industry as a stabilizing, gelling and thickening agent, as well as a fat substitute, especially in dairy products.^14,15^ Additionally, studies on carrageenan's pharmacokinetics and tissue distribution demonstrate its absorption, metabolism, and accumulation in organs like the liver and kidney, laying the foundation for its pharmaceutical applications.^16^ However, it is crucial to consider the potential harmful effects of carrageenan on intestinal inflammation, emphasizing the importance of understanding its dual nature for therapeutic purposes.^17,18^

### 1.1 Purpose of study

Carrageenan, a sulphated polysaccharide from red seaweeds, has been extensively investigated in many published articles and reviews. The literature, however, is inclined to emphasize either industrial uses, biomedical attributes, or nutritional implications, tending to overlook a multidisciplinary approach that encompasses its wider applications. Whereas various reviews have scrutinized its function in food stabilization, drug formulation, and potential hazards to human health, no one have holistically addressed the role of carrageenan towards environmental sustainability, especially wastewater treatment. The current review fills the lacuna by synthesizing carrageenan's structural attributes, functional diversifications, and multi-domain applications in environmental, food, and biomedical sciences. In contrast with earlier reviews focused either on its biomedical promise, for instance, in drug delivery and wound healing, or on its contentious impacts on gastrointestinal well-being, this research provides a more balanced strategy. It addresses in detail how structural adjustments, including molecular weight decrease and nanocomposite structures, strengthen the potency of carrageenan for selective drug delivery, bioimaging, and regenerative medicine. This review goes beyond conventional rhetoric by assessing its applications in environmental cleanup, noting its success in the removal of heavy metals, dye adsorption, and industrial wastewater treatment. Although there is huge research on carrageenan's food uses, mostly revolving around its use as a stabilizer, gelling agent and texture modifier, this review extends beyond that by exploring its applications in biodegradable food packaging and edible coatings. The convergence of carrageenan with green food technology is an important area that is underrepresented in current literature. By discussing both the advantages and disadvantages of carrageenan-based products in food packaging and preservation, this research offers useful information for the development of environmentally friendly substitutes for synthetic additives. Safety concerns over carrageenan, especially its association with inflammatory bowel disease and other gastrointestinal symptoms, have been debated in various reports. Yet, most of these reports offer polarized opinions, either deeming it unsafe or denying concerns altogether. This review critically analyses existing data with the understanding that there are both health risks posed and modification possibilities that can nullify negative outcomes. Through incorporating regulatory insights as well as novel developments in the biomedical applications of carrageenan, this assessment guarantees a complete and balanced examination. Due to the fragmented form of existing studies, this review is unique because it provides an interdisciplinary analysis tying together carrageenan's utilization in different sectors. Not only does it integrate information from diverse fields but also accentuates evolving developments, like the use of carrageenan in nanomaterials for improved drug delivery and in the creation of next-generation biocompatible scaffolds. This integrative approach makes the study a valuable contribution to academics and industry alike, informing future research into innovative and sustainable uses of carrageenan.

### 1.2 Sources

Carrageenan is sourced from red seaweed of the Rhodophyceae family by extraction, typically from genera such as Eucheuma, Agardhiella, Solieria, Iridaea, Cripus, Hypnea, Chondrus, Sarconema, Gigartina, and Stellate.^19–24^ Euchema and Kappaphycus seaweeds are the most widely found seaweeds in Malaysia and Southeast Asia.^25^

## 2. Structure and properties

**Fig. 1:**
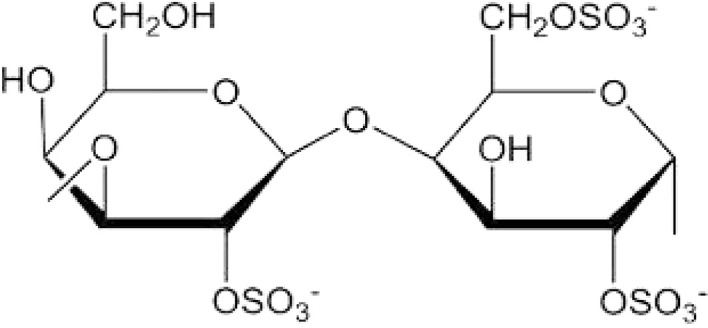
Structure of carrageenan.

Carrageenan, a sulfated polysaccharide derived from marine red algae, exhibits versatile properties and structures crucial for various applications.^26^ Research indicates that carrageenans with a 3,6-anhydrous bridge adopt compact helical structures, while those without the bridge remain as extended helices, contrary to the ‘coil-to-helix’ paradigm.^27^ These polysaccharides are composed of long, linear polymer chains with fundamental structural units comprising two galactose moieties, one with a β-linkage at the 3-position and the other with an α-linkage at the 4-position or a 3,6-anhydro-α-galactopyranose unit, which can vary in the number and position of sulphate groups attached to the galactose units within the disaccharide repeating units.^28,29^ The chemical structure of carrageenan is given below in Fig. 1.

Additionally, the degradation of high molecular weight κ-carrageenan into low molecular weight derivatives enhances its antibacterial activity, with structural characteristics like molecular weight, 3,6-anhydro-d-galactose content, and sulphate groups influencing this activity.^30^

Furthermore, carrageenan's biodegradability, nontoxicity, and water solubility make it a promising material for food packaging applications, with modifications improving its mechanical, thermal, and antibacterial properties.^31^ These insights highlight carrageenan's potential in diverse fields, from wound healing to food preservation, showcasing its significance in advancing biocompatible and sustainable solutions.^32^Table 1 highlights the comparison between structural and chemical properties and applications of kappa and iota carrageenan.

**Table 1:** Highlighting the comparison between structural and chemical properties and applications of kappa and iota carrageenan

Property	Kappa-carrageenan	Iota-carrageenan	References
Chemical structure	Alternating 3-linked β-d-galactopyranose and 4-linked 3,6-anhydro-α-d-galactopyranose; lower sulfate content (∼25–30%)	Similar backbone but higher sulfate content (∼28–30%) with sulfate groups at C-4 of the α-d-galactose unit	28 and 33
Gelation ions	Forms strong, rigid gels in the presence of potassium ions (K^+^)	Form soft, elastic gels in the presence of calcium ions (Ca^2+^)	34 and 35
Gel strength & texture	Strong, brittle, and rigid gels with high water retention	Soft, flexible, elastic gels with freeze-thaw stability and less syneresis	36 and 37
Gelation mechanism	Helix aggregation is promoted by cation binding, forming a 3D network; gelation occurs over a wider ionic strength range	Two-step gelation with more strain-softening; gels sustain larger strains before yielding	38 and 39
Thermal stability	Higher gel melting temperature; gels are thermally more stable	Lower melting temperature but better freeze-thaw stability	40–43
Solubility	Soluble in hot water; forms gels upon cooling	Soluble in hot water; gels are more elastic and less prone to syneresis	44 and 45
Applications	Used for firm texture in dairy, meat products, and food stabilizers	Used where elasticity and flexibility are needed, *e.g.*, in soft gels and pharmaceutical formulations	15 and 46–49

Carrageenan, a naturally occurring polysaccharide sourced from red seaweed and trialent ions Eu^3+^, Nd^3+^, Sm^3^ displays a range of optical behaviors especially glasses and luminescent complexes are highly valuable for applications in fields such as laser technology, optical amplifiers, magnetic resonance imaging (MRI), and emerging quantum systems that can be adjusted through chemical modifications or the incorporation of different additives. These tunable properties are essential for its application in fields such as food packaging, edible films, and optical sensing technologies. By altering its molecular structure or blending it with compatible materials, carrageenan's functional performance can be enhanced for targeted uses. Beyond its optical potential, carrageenan also exhibits favorable electrical characteristics, making it a strong candidate for use in flexible, eco-friendly electronic devices. As a substrate, it provides excellent optical clarity, with light transmittance exceeding 90% at 550 nm, supporting its role in optoelectronic systems. Electrically, carrageenan-based resistive switching devices demonstrate consistent performance, characterized by high ON/OFF current ratios (greater than 10^6^), stable retention exceeding 10^4^ seconds, and resilience to repeated mechanical deformation. The underlying switching behavior results from the formation and disruption of conductive pathways consisting of both silver and carbon, driven by ion migration and redox processes involving carrageenan's reactive functional groups. Furthermore, these devices remain operational under everyday environmental conditions and can be applied directly to skin, emphasizing their suitability for wearable technologies and biodegradable transient electronics^50,51^

### 2.1 Types of carrageenan

Carrageenan mainly consists of 3 types, distinguished by their degree of sulfation.

**Fig. 2:**
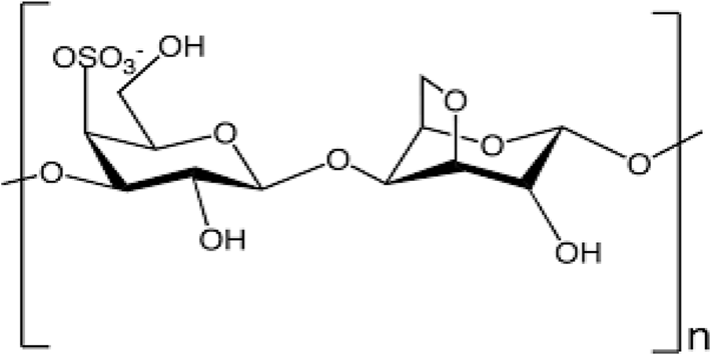
Chemical structure of kappa (κ)-carrageenan^54^

**Fig. 3:**
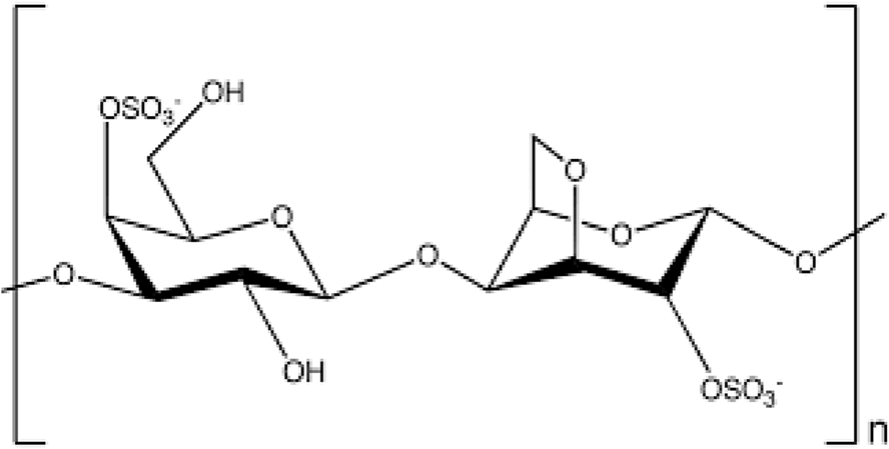
Chemical structure of iota (ι)-carrageenan.^61^

(i) Kappa (κ)-carrageenan is a specific type of carrageenan that exhibits a structural composition characterized by alternating 3-linked β-d-galactose 4-sulfate and 4-linked 6-anhydro-α-galactopyranose units within its repeating disaccharide moieties. This arrangement results in the presence of one negative charge per repeating disaccharide unit, which contributes to the unique physicochemical properties and functional characteristics of κ-carrageenan.^18,24,52,53^ The chemical structure of k-carrageenan is given below in Fig. 2.

(ii) Iota (ι)-carrageenan consists of two sulfate groups in each repeating disaccharide moiety.^28,55^ Iota and kappa carrageenan have similar properties. In aqueous solutions, both κ- and ι-carrageenan experience thermoreversible conformational changes at higher temperatures. The gelling and network-forming properties of certain carrageenan's, such as iota (ι)- and kappa (κ)-types, are influenced by their structural characteristics and interactions with cations in the surrounding environment, where at lower temperatures, they form robust networks through interactions involving sulfate groups and the 3,6-anhydro-d-galactopyranosyl ring, while their gelation behaviour is also strongly affected by the concentration and type of cations, such as K+ and Ca^2+^, as well as the overall biopolymer concentration, highlighting the versatility and tailor ability of these sulfated polysaccharides in various applications where controlled gelation and network formation are desirable.^56–58^ Furthermore, various salts affect the phase transitions and gelling of ι- and κ-carrageenan gels differently. Studies have shown that κ-carrageenan forms stronger gels in the presence of KCl compared to other salts such as NaCl, MgCl_2_, LiCl, SrCl_2_, and CaCl_2_.^59^ Additionally, the gel–sol transition temperatures of κ-carrageenan are strongly influenced by the concentrations of KCl, NaCl, and CaCl2.^60^ The chemical structure of iota carrageenan is given in Fig. 3.

(iii) Lambda carrageenan consists of 3 sulfate groups per disaccharide unit, but there is no 3, 6-anhydride bridge, contrary to other types.^55^ λ-Carrageenan shows random conformations at all temperatures and is unable to form a gel.^56^ The latest studies revealed that gelation in λ-carrageenan can be induced by using trivalent ions. This aspect can increase the future utilization of λ-carrageenan.^62^ Recent studies revealed that through the incorporation of trivalent ions, a highly sulphated and flexible structure can be achieved, which facilitates gel formation in carrageenan as trivalent ions possess high charge density, allowing them to neutralize sulphate groups in carrageenan. This balancing promotes strong interaction and forms a stable three-dimensional gel with enhanced thermal and mechanical stability. The ability of trivalent ions to induce gelation expands their application potential as a viscosifier or stabilizer. This development opens new avenues, including food technology, pharmaceuticals and biomedical engineering, controlled drug delivery, wound healing, and tissue engineering benefit greatly from the formation of these gels.

Moreover, the selection of trivalent ions tailored gel properties enables precise control over thermal behavior, gelation kinetics and mechanical strength. This provides an advantage in designing advanced biomaterials for industrial or medical purposes.^62,63^

**Fig. 4:**
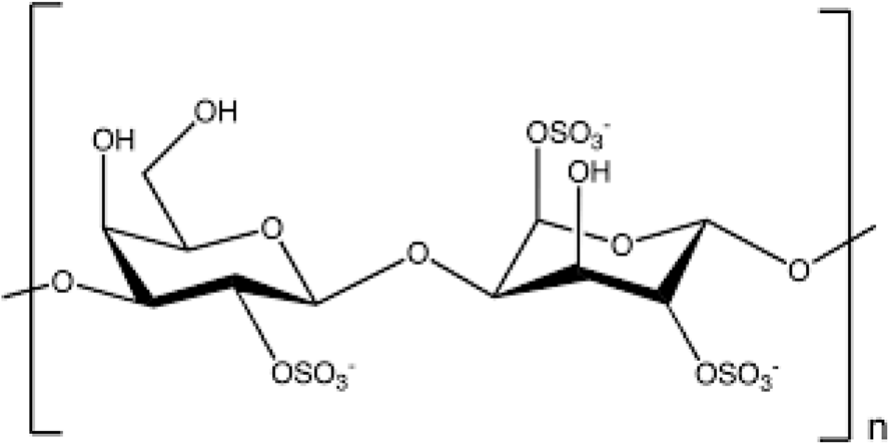
Chemical structure of lambda carrageenan.

The chemical structure of lambda carrageenan is given in Fig. 4.

## 3. Disadvantages of carrageenan

Recent research has highlighted several disadvantages of carrageenan, a commonly used food additive. Studies have shown that carrageenan, particularly those with random coil conformations, can have adverse effects on gastrointestinal health by aggravating intestinal inflammation.^64–66^ Carrageenan has been linked to changes in the intestinal microflora, leading to a reduction in beneficial bacteria like Bifidobacteria and an increase in harmful bacteria such as Akkermansia muciniphila, ultimately compromising the integrity of the intestinal membrane and mucin layer.^10^ Furthermore, carrageenan has been associated with the activation of pro-inflammatory pathways, such as the NF-kB pathway, through interactions with TLR4 receptors and alterations in macrophage activity, leading to inflammatory reactions in the gut.^65^ Carrageenan may cause inflammation in the gut, especially in people who already have digestive issues. However, not everyone reacts in the same way. Many factors cause differences that vary from individual to individual, such as carrageenan type used, diet, gut health and make-up of persons gut bacteria. Because of these variations, general recommendations may not be suitable for everyone. The guidance on carrageenan should be personalized, especially for those who may be more sensitive. This approach helps avoid any doubt and ensures that dietary advice is based on individual health needs and risks. Several carrageenan-based composite systems have been investigated for toxicity, with various outcomes depending on composition, intended use, and exposure duration. A carrageenan/agarose composite sponge used for biomedical applications showed low acute cytotoxicity *in vitro*. When fibroblast (NIH-3T3) and macrophage (RAW264.7) cells were exposed to the sponge's leachate at high concentrations (2100 μg mL^−1^), cell proliferation remained above 80% during the first two days, indicating low toxicity. A carrageenan/agarose-based sponge composite showed low acute toxicity *in vitro* studies, but when the sponge's leachate was exposed to fibroblast (NIH-3T3) and macrophage at high concentrations, cell proliferation remained above 80% during the first 2 days, which indicated low toxicity. However, fibroblast viability dropped to 61%, showing delayed cytotoxic effects, possibly due to degradation products or leachate components. This indicates that longer exposure may reveal toxicity concerns despite initial desirable results.^67^

A comprehensive research study on semi-refined food graded carrageenan (E407a) showed systematic local and toxic effects in 2 weeks of oral exposure. Historical examination revealed infiltration of macrophages (CD68+ cells) into the small intestine, leading to inflammation in the intestine. Rats also showed a high level of blood inflammation and high cell death. When rats were exposed to F407a in the lab, nothing bad happened, suggesting that inflammation in the body causes immune dysfunctions. This suggests that carrageenan caused toxicity and inflammation, so more research is needed to address cytotoxicity.^68^ Another experimental study investigated the cytotoxic effect of oxidized degraded product of κ-carrageenan, *i.e.*, κ-poligeenan (κ-CODP), on human colonic epithelial Caco-2 cells. Significant toxicity was found at concentrations above 40 μg mL^−1^, mainly due to ROS production. It was concluded that κ-CODP facilitated apoptosis as well as inflammation of colonic epithelial cells through the ROS pathway, indicating its potential toxicity in drug and food processing.^69^Table 2 highlights the disadvantages of carrageenan, their potential impacts, and the food sector affected by these issues.

**Table 2:** Highlighting the disadvantages of carrageenan, their potential impacts, and the food sector affected by these issues

Disadvantage	Description	Potential impact
Potential carcinogenicity	Possible link to increased risk of gastrointestinal cancers (mainly with degraded form)^70^	Regulatory scrutiny; consumer mistrust
Allergic reactions	Can cause allergic responses in some individuals^71^	Limited applicability; labelling requirements
Nutrient absorption interference	May hinder the absorption of certain nutrients^72^	Nutritional concerns in food products gut discomfort
Environmental concerns	Overharvesting can disrupt marine ecosystems^73^	Sustainability issues, supply chain challenges
Processing challenges	Sensitive to pH, temperature, and ionic conditions^74^	Increased production costs; inconsistent product quality
Stability issues	Potential for syneresis and texture changes during storage^75^	Reduced shelf life; product quality concerns
Consumer perception	Growing negative perception as a food additive^76^	Demand for carrageenan-free products; marketing challenges
Source material variability	Properties can vary based on seaweed species and conditions^77^	Inconsistent product performance, quality control issues
Limited functionality in acidic conditions	Reduced effectiveness in highly acidic environments^78^	Formulation limitations in certain products
Potential for contamination	Risk of heavy metal or pollutant contamination^79^	Increased quality control costs, safety concerns

These findings emphasize the need for personalized guidance on carrageenan intake based on individuals' health status and further research to understand the impact of carrageenan on gastrointestinal health.

## 4. Applications of carrageenan in wastewater treatment

**Fig. 5:**
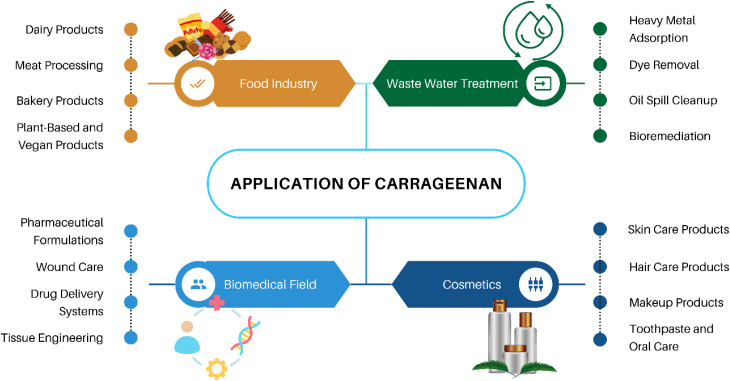
Application of carrageenan in different industries.

**Fig. 6:**
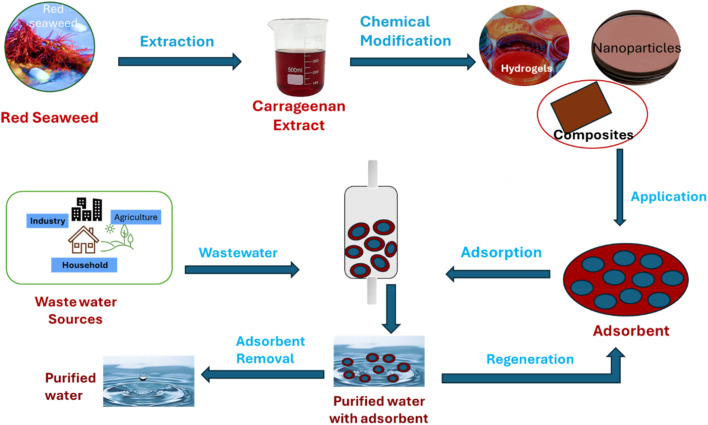
Pathway from extraction to application of carrageenan in wastewater treatment.

Carrageenan, a biopolymer, has shown significant potential in wastewater treatment applications, as shown in Fig. 5. Studies have demonstrated the successful development of carrageenan-based adsorbents for the removal of cationic dyes from wastewater.^80–82^ By utilizing carrageenan in combination with other materials like magnetic nanoparticles and graphene oxide, researchers have created adsorbents with high adsorption capacities and excellent stability, making them effective in treating dye-contaminated water. The synthesized carrageenan-based materials exhibited impressive adsorption capacities for various cationic dyes, such as methylene blue, malachite green, and safranin T, showcasing their efficiency in wastewater treatment processes.^83,84^ Additionally, the recyclability of these carrageenan-based adsorbents over multiple cycles further highlights their suitability for sustainable water treatment applications. The general pathway from source to application of carrageenan-based adsorbents is shown in Fig. 6. This diagram illustrates a sustainable process for wastewater treatment using red seaweed-derived carrageenan-based adsorbents. The process begins with the extraction of carrageenan from red seaweed, followed by chemical modification to produce advanced materials such as hydrogels, nanoparticles, and composites. These modified materials are then applied as adsorbents to remove contaminants from wastewater sourced from households, agriculture, and industry. During the adsorption phase, pollutants adhere to the adsorbent, resulting in purified water. The adsorbent is subsequently separated from the treated water and regenerated for reuse, making the process both efficient and environmentally friendly.

### 4.1 Heavy metals

**Fig. 7:**
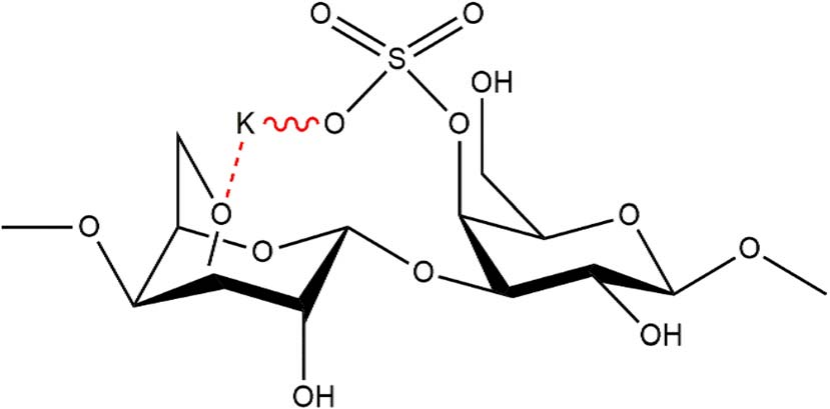
Intramolecular cation-selective bridging in κ-carrageenan in aqueous solution at low temperatures involves 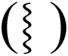 ionic bonding and (- - - -) electrostatic attractive forces.

A group of researchers prepared a composite hydrogel by mixing kappa-carrageenan, *i.e.*, sulphate-based plant polysaccharides Vallin and DI water, followed by a gravity filtration step. Researchers employed this gel for wastewater treatment. KC-Vallin showed an advantage over commercial filtration media as it has efficient wastewater treatment, better permeability, less energy consumption, and pocket-friendly infrastructure. Vallin has biocidal properties in addition to others. This system rejected heavy metals like Pb, Ba, Al, Cd and Cr. This system rejected about 77% of the organic carbon content from the Leachate Water.^85^ The intramolecular cation-selective bridging in κ-carrageenan in aqueous solution at low temperatures is shown in Fig. 7.

**Fig. 8:**
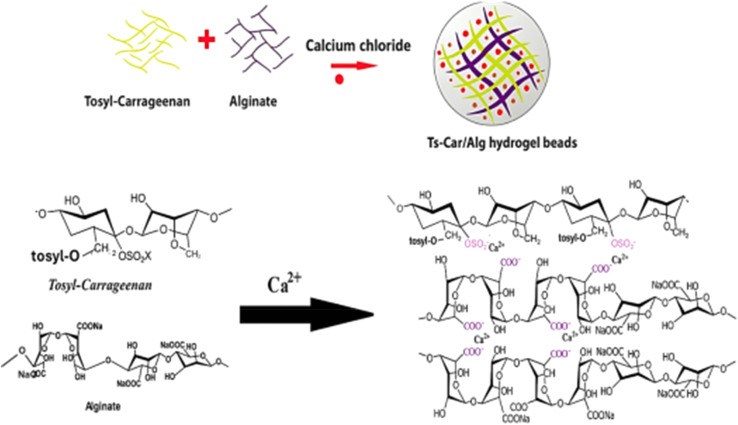
Tosyl-carrageenan system for the removal of heavy metals from wastewater, reprint with permission^90^

In another study, novel buckypaper (BP) membranes were fabricated from multi-walled carbon nanotubes (MWCNTs) using a vacuum filtration method, and the ability of chitosan and carrageenan biopolymers to enhance MWCNT dispersion in water was investigated, both individually and in combination. For the first time, the MWCNTs combined with chitosan and carrageenan showed excellent mechanical strength and high heavy metal rejection, achieving 94% copper and 91% lead removal at an applied pressure of one bar.^86^ In this study, a κ-carrageenan/cellulose (κ-CG/CL) hydrogel was synthesized using a simple one-step method for efficient removal of Pb^2+^ ions from aqueous solutions. The hydrogel's functional groups and crystalline structure were characterized by FTIR and XRD, while SEM and BET analyses confirmed its porous surface morphology, with pore sizes ranging from 1–10 μm. The κ-CG/CL hydrogel demonstrated strong Pb^2+^ removal capabilities, with adsorption performance evaluated under varying pH levels and contact times, fitting well with multiple kinetic and isotherm models, including Langmuir and Freundlich. The hydrogel's maximum adsorption capacity, as per the Freundlich model, was 486 ± 28.5 mg g^−1^, achieving over 79% Pb^2+^ removal efficiency after eight reuse cycles. These results indicate that κ-CG/CL hydrogels hold promising potential for removing and recycling heavy metal ions from water.^87^ Another research study worked and made out nanocomposite-based magnetic hydrogel using k-carrageenan, activated carbon and acrylic acid. Synthesized hydrogel was applied to remove various heavy metal ions such as Ni^+2^, Cu^+2^, Cd^+2^ and Cu^+2^. The adsorption capacity by the Langmuir model was found to be 156.25, 294.11, 454.54, and 285.71 mg g^−1^ for Ni^2+^, Co^2+^, Cd^2+^, and Cu^2+^, respectively. They obtained desired adsorption results, and reusability was observed to be 60% after 6 cycles.^88^ Innovative cross-linked tosyl-carrageenan/alginate beads have been produced and characterized with structural analysis being conducted by means of XRD, SEM, FTIR, and EDX analyses, as well as assessment through batch experiments regarding the adsorption behaviour. Material exhibited its best adsorption ability at a pH of 5.3 after 120 min with maximum capacities of 74 mg g^−1^ for Pb^+2^ ion. Kinetic and isotherm studies reveal the pseudo-second order mechanism as well as fitting the Freundlich models, indicating that such beads are a suitable candidate for reusability in heavy metal remediation processes.^89^ The chemistry behind this system is shown in Fig. 8.

### 4.2 Dyes

Chemists prepared Tripple Bio Composite (TBC) beads by chemical precipitation method using NHAP, calcium alginate and kappa-carrageenan. Formulated TBC exhibited biodegradability, hydrophilicity, nanotoxicity and adsorption properties. Batch adsorption of methylene blue was executed. XRD Analysis confirmed the crystalline nature of NHAP. Swelling studies revealed NHAP had lower hydrophilicity, while KPC(Kappa/Carrageenan/Nanohydroxyapatite) and TBC had minimal swelling. These factors give an indication of TBC as an efficient adsorbent by controlling water adsorption. The maximum adsorption capacity exhibited by TBC for methylene blue was 529.1 mg g^−1^. Hydrochloric acid was found to be the most effective desorbing agent (86% desorption efficiency).^91^ In another studyresearchers crafted green adsorbent, *i.e.* mountain Apricot Shell Melanin (Mass/Mel), graft copolymerized with k-carrageenan. These formulated hydrogel beads showed a uniform spherical and 3-D porous structure under SEM. TGA analysis revealed low weight loss, indicating thermal stability. According to the Langmuir adsorption model, the maximum adsorption capacities of methylene blue and malachite green were 48.63 mg g^−1^ and 37.8 respectively, under an optimum pH of 6–7, 150 min contact time and 60 mg adsorbent dose.^92^ Jincheng Yu and Co. developed biomass-based aerogel from polydopamine-coated pomelo peel, polyethyleneimine and k-carrageenan (PPEKC) for the removal of cationic and anionic dyes. SEM results revealed that PPEKC aerogel had a hierarchical microporous honeycomb-type cell structure. Adsorption of congo red and methylene blue was endothermic and spontaneous. Due to the pH-tuneable surface, at low pH (positively charged PPKEC), maximum adsorption efficiency of negative dyes occurred.^93^ A membrane made up of PVA having 3 types of carrageenan (PVA/carrageenan) has been used for the removal of methylene blue. Experimentation showed PVA/k-carrageenan membrane developed 147.8 mg g^−1^ adsorption capacity. Crafted Membrane exhibited 98% removal efficiency as well as compared to pure PVA, 6.3%. Among the various carrageenan-based adsorbent systems, Triple Bio Composite (TBC) beads showed the highest adsorption capacity (529.1 mg g^−1^) for methylene blue, due to the synergistic effect of NHAP, calcium alginate, and kappa-carrageenan, along with their controlled swelling behavior. In contrast, the Mass/Mel-kC hydrogel beads exhibited moderate capacities but offered advantages like thermal stability and eco-friendly preparation. The PPEKC aerogel stood out for its pH-responsive surface, making it versatile for both cationic and anionic dye removal, although adsorption capacities were not explicitly reported. The PVA/k-carrageenan membrane provided a balanced approach, achieving 98% removal efficiency with good capacity (147.8 mg g^−1^), suitable for membrane filtration systems.

Overall, TBC beads are the most promising candidate for high-capacity dye adsorption, especially where desorption and material reusability are important. However, depending on the specific application, prioritizing removal efficiency, structural properties, or environmental compatibility, each system offers unique advantages.

**Fig. 9:**
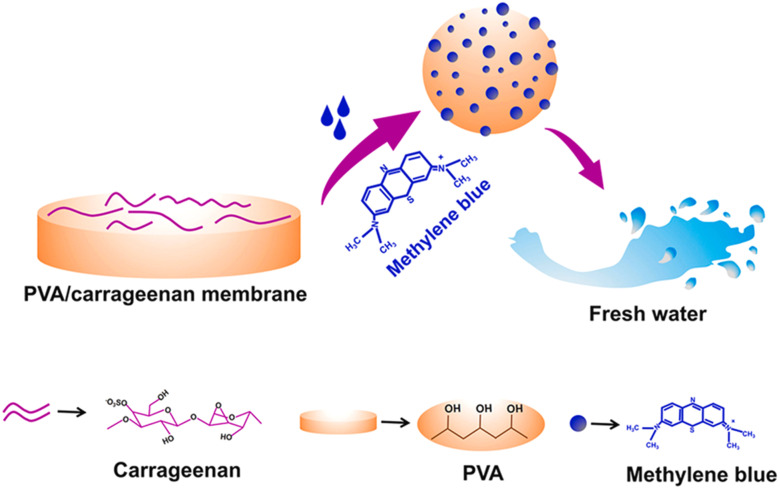
PVA/carrageenan membranes system for the removal of methylene blue from water. Reprinted with permission from Sabarish Radoor *et al.*, *Chemosphere*, 2024, **350**, 140990. © 2024 Elsevier.^94^

The mechanism of the membrane for the degradation of methylene blue involves the combination of hydrogen bonds and electrostatic interactions. Cationic methylene blue molecules and the negatively charged sulphate group molecules found in carrageenan play a vital role in the separation of dye from solution and produce purified water. The scheme for the degradation of methylene blue is shown in Fig. 9.^94^ Furthermore, researchers first time utilized hydrochar-derived activated carbon to enhance the adsorption capacity of carrageenan. These Hydrogel beads effectively removed methylene blue. Swelling studies revealed that water holding capacity increased with an increase in the content of activated carbon.^95^

**Fig. 10:**
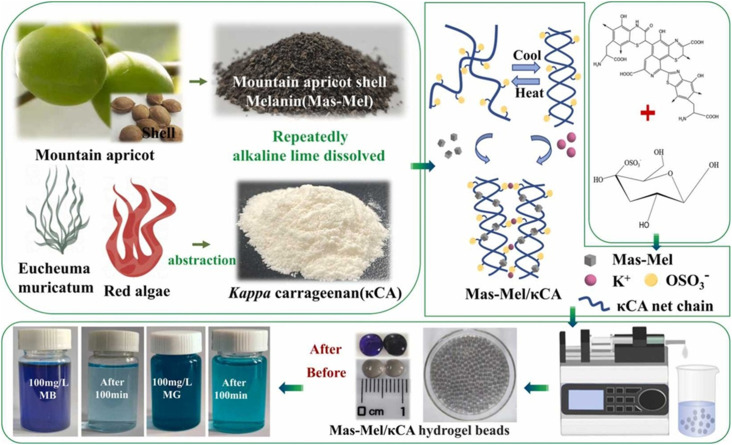
Synthetic strategy for Mas-Mel/k-carrageenan hydrogel beads. Reprinted with permission from Rui Hao *et al.*, *Chemical Engineering Research and Design*, 2023, **199**, 1–10. © 2023 Elsevier.^92^

Khushboo and Co. developed nano catalytic platform (BTCSNC) by creating k-carrageenan and silver nano catalyst (CSNS) immobilized on bentonite sheets for azo dyes degradation. Immobilization step enhanced degradation efficiency by 3–4 folds.^96^ Duman and Co synthesized a hydrogel made up of cross-linked agar/k-carrageenan and utilized it in the adsorption of methylene blue. Adsorption process obeyed the Langmuir Isotherm Models and pseudo-2nd order kinetics. The adsorption capacity of hydrogel increases as pH increases from 1–7. The maximum adsorption capacity obtained was 242.3 mg g^−1^ at pH 7 and temp 35 °C.^5^ Herbicides have been widely used in agricultural fields, leading to their settling in water reservoirs as well as harm to aquatic bodies. In view of this, experimenters utilized carrageenan and developed an effective adsorbent based on biomass for the elimination of cationic/anionic dyes, herbicides, as well as metal ions from wastewater. They made KC/PEI/GTE and achieved efficient results up to 93%. The magic forces behind these adsorption capacities were reported to be hydrogen bonding and electrostatic interactions.^97^ Another composite k-carrageenan-grafted poly having multi-walled CNT composite was synthesized using a grafting polymerization mechanism. Novel material was used in removing safranin-O dye from water. Due to the porous nature and various functionalities, an adsorption capacity of 10.71 mg g^−1^ towards dye removal was achieved after 90 minutes of batch adsorption.^98^ Using an *ex situ* fabrication approach, researchers synthesized k carrageenan/polyacrylamide/magnetite nanocomposites and checked their effectiveness on the eradication of methylene blue and rhodamine. Maximum adsorption capacities above 200 mg g^−1^ were obtained, and the reaction was observed to be endothermic and followed pseudo-first-order kinetics. In addition to good removal efficiency, it also showed good reusability.^99^ This work used bilberry kernel shells to isolate Mountain apricot shell melanin (Mas-Mel), which was κ-carrageenan-grafted to obtain hydrogel beads for cationic dye adsorption. The best adsorption of methylene blue and malachite green was observed at pH 6–7, adhering to pseudo-second-order kinetics, with capacities of 48.63 mg g^−1^ and 37.84 mg g^−1^. The hydrogel beads proved to be resilient, with 50% efficiency persisting after three prolonged reuse cycles, presenting a promising green approach to wastewater treatment^92^ The process cycle of Mas-Mel/k-carrageenan formation is shown in Fig. 10. Table 3 shows a comprehensive overview of carrageenan applications in wastewater treatment, highlighting mechanisms, advantages, and potential challenges.

**Table 3:** Comprehensive overview of carrageenan applications in wastewater treatment, highlighting mechanisms, advantages, and potential challenges

Application area	Description	Mechanism	Advantages	References
Flocculation	Carrageenan acts as a flocculant in removing suspended solids from wastewater. 0.5–2% κ-carrageenan	Forms aggregates with particulates, enhancing sedimentation	Effective in reducing turbidity; eco-friendly	100
Heavy metal removal	Binds to heavy metals in wastewater, facilitating their removal. Use 1–3% κ-carrageenan	Metal ions interact with sulfate groups in carrageenan	Reduces toxicity; can recover metals for recycling	101
Nutrient recovery	Carrageenan aids in the recovery of nutrients like nitrogen and phosphorus. 1–2% κ- and ι-carrageenan	Forms complexes with nutrients, improving bioavailability	Enhances nutrient recycling; supports sustainable practices	102 and 103
Biodegradable adsorbent	Used as an adsorbent for organic pollutants and dyes. 0.5–2% λ-carrageenan	Adsorbs contaminants *via* electrostatic and hydrophobic interactions	Biodegradable and non-toxic; efficient in colour removal	84
Biofilm formation	Supports biofilm development in bioreactors for enhanced biodegradation. 0.5–1% carrageenan is used	Provides a surface for microbial colonization and growth	Improves treatment efficiency; increases microbial diversity	104
Sludge management	Carrageenan can modify sludge properties for easier handling. 1–2% κ-carrageenan	Enhances dewaterability and reduces volume	Lowers disposal costs; improves operational efficiency	105
Microbial growth stimulation	Enhances microbial activity in treatment systems. 0.5–1% carrageenan	Provides nutrients and structural support for microorganisms	Increases treatment rates; supports diverse microbial communities	106
Membrane filtration	Used in conjunction with membranes to reduce fouling. 0.3–1% κ-carrageenan	Forms a protective layer, minimizing direct contact with membranes	Extends membrane life; reduces cleaning frequency	107

### 4.3 Drugs

**Fig. 11:**
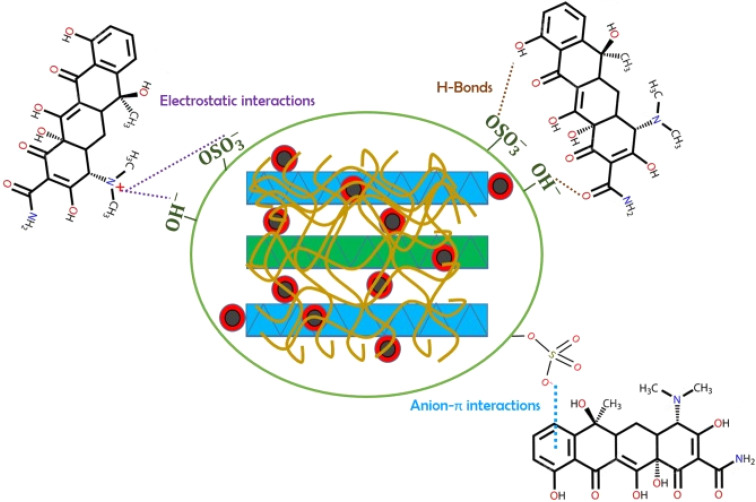
Interaction mechanism of tetracycline and FACM adsorbent. Reprinted with permission from Amirreza Ahmadpour *et al.*, *Inorganic Chemistry Communications*, 2023, **156**, 111274.© 2023 Elsevier.^108^

Researchers designed conjugated montmorillonite clay and k-carrageenan-based magnetic adsorbents for the removal of tetracycline from aqueous media. The adsorbent obtained had a crystalline, rough surface with a specific area of 57.5 m^2^ g^−1^ and a 3-D structure of micro- and mesopores. Results showed that it worked best in a pH range of 4–11 and at temp 45 °C 80.28 mg g^−1^, adsorption capacity was obtained. It is higher than that of other polymeric adsorbents. Thermodynamic data revealed adsorption process is spontaneous and endothermic. Regeneration was obtained through HCl. The interaction mechanism between the tetracycline drug and the FACM adsorbent is shown in Fig. 11.^108^ Furthermore, another novel material, *i.e.* nanocomposites made up of carrageenan-grafted polyacrylamide having Fe_3_O_4_ nanoparticles and incorporated MOF, was used for the removal of drugs from aqueous solution. This eco-friendly 3-d hydrogel effectively entrapped drug particles. Maximum adsorption capacity of 2000 and 1666.667 mg g^−1^ obtained for Levofloxacin and Cefixime, respectively.^109^

### 4.4 Organic compounds

The mentioned study developed a biopolymer adsorbent comprised of chitosan/k-carrageenan hydrogel for the removal of phenol from wastewater. The hydrogel showed a remarkable adsorption performance of 80% uptake. Use of k-carrageenan enhanced adsorption capacity as well as stability by cross-linking.^110^

## 5. Biomedical applications

**Fig. 12:**
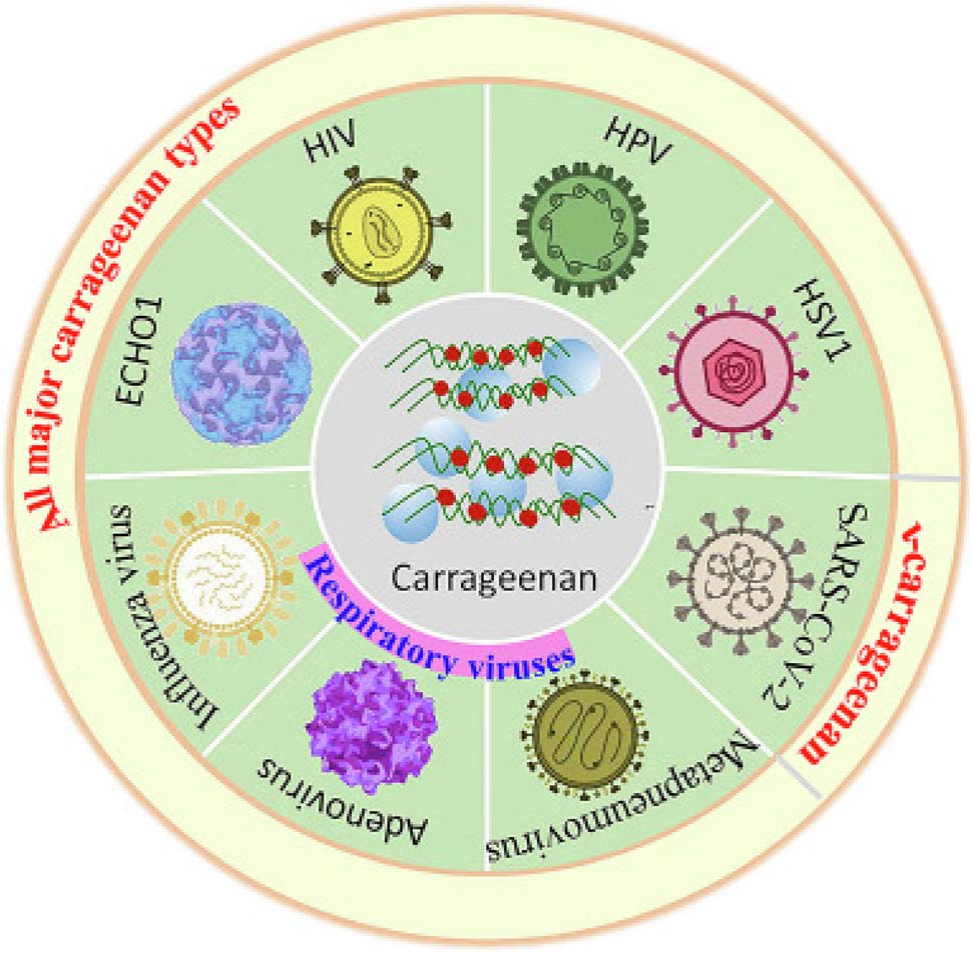
Anti-viral activities of different carrageenan types. Reprinted with permission from Fang Liu. *et al.*, *International Journal of Biological Macromolecules*, 2023, **235**, 123787.© 2023 Elsevier.^112^

Carrageenan isolated from red algae has attained more attention because of its potential applications in biomedical sciences. It is favourable for biomedical engineering applications due to its outstanding biocompatibility, non-toxicity and biodegradability in drug delivery systems, tissue regeneration and wound healing. These characteristics allow materials based on carrageenan to function as scaffolds or carriers that minimise adverse host reactions while integrating with biological environments.^111–113^ Carrageenan is noteworthy for its intrinsic bioactivity, which includes antiviral, antibacterial, and anticancer qualities. The carrageenan antiviral mechanism is very well documented. Multiple studies demonstrate that carrageenan interferes with the viral life cycle at the initial attachment phase. It has been noted that carrageenan (lambda _CG) binds to viral particles and forms non-reversible complexes that obscure important glycoprotein structures on the viral envelope, preventing the virus from adhering to host cell receptors.^114^ In contrast to the intracellular effect of various traditional antiviral medications. This early-stage interference offers a prophylactic mechanism. Different types of carrageenan have antiviral activities against different viral families according to comparative analysis across studies, although the strength and spectrum of this activity seem to be controlled by structural variables such as molecular weight and sulfate concentration.^113^ Numerous carrageenan types are capable of inhibiting multiple viruses.^112^Fig. 12 illustrates how carrageenan molecules engage with viral surface proteins to inhibit cellular entry. The figure also highlights the structural variations among carrageenan types and their correlation with antiviral efficacy.

However, there are certain issues with its clinical translation, concerns about gastrointestinal have been raised when it comes to long-term use. Carrageenan safety has been called into question after many studies connected its exposure to disruptions in the gut Flora and low-grade intestinal inflammation. These results stand in contrast to earlier *in vitro* research, which mainly assessed carrageenan in controlled settings devoid of the complexity of living systems. These disparities highlight the need for more rigorous, long-term *in vivo* studies to completely understand the immunological and metabolic effects of materials based on carrageenan.^64,115^ These findings contrast with previous studies, which primarily focused on its *in vitro* safety profile, potentially underestimating *in vivo* immunological interactions. This disparity emphasizes the necessity of more thorough, longitudinal animal and clinical research to evaluate its systematic effects over time. Despite these challenges, ongoing research aims to harness the exceptional properties of carrageenan to develop innovative solutions for tissue repair, disease intervention, and wound healing, showcasing its transformative role in advancing biomedical applications. Table 4 shows the biomedical application of carrageenan with the mechanism of action and advantages.

**Table 4:** Biomedical application of carrageenan with mechanism of action and advantages

Application area	Description	Mechanism	Advantages	Preferred amount/type	References
Drug delivery systems	Carrageenan is used as a matrix for controlled drug release	Form hydrogels that encapsulate drugs, allowing sustained release	Biocompatible; can improve drug solubility and stability	1–3% κ-carrageenan	124 and 125
Tissue engineering	Serves as a scaffold for cell growth and tissue regeneration	Provides a supportive environment for cell adhesion and proliferation	Biodegradable; mimics natural extracellular matrix	0.5–2% ι-carrageenan	126
Wound healing	Carrageenan-based dressings promote healing and reduce infection	Forms a gel that maintains moisture and protects the wound site	Enhances the healing process; reduces scarring	1–2% λ-carrageenan	111 and 127–129
Antimicrobial applications	Exhibits antimicrobial properties against various pathogens	Interacts with microbial cell membranes, disrupting their function	It can be used in coatings or formulations to prevent infection	0.5–1% κ-carrageenan	130–132
Vaccines and immunology	Carrageenan can be used as an adjuvant to enhance vaccine efficacy	Stimulates immune response, improving the effectiveness of vaccines	Safe and natural; enhances immunogenicity	0.1–0.5% carrageenan	133–135
Oral health products	Incorporated in mouthwashes and gels for oral care	Provides a soothing effect and helps in the formation of protective films	Reduces inflammation; improves mucosal healing	0.5–1% λ-carrageenan	136–138
Bioimaging	Carrageenan is used in imaging agents for enhanced contrast	Increases viscosity and stabilizes imaging compounds	Non-toxic; improves visualization of tissues	1–2% κ-carrageenan	53, 139 and 140
Hydrogels for cell encapsulation	Used to encapsulate cells for therapy and research applications	Form stable hydrogels that protect cells from immune response	Promotes cell viability; customizable properties	1–3% mixture of κ- and ι-carrageenan	141 and 142
Cosmetic applications	Used in formulations for skin care and anti-ageing products	Provides viscosity and stability while enhancing skin hydration	Biocompatible; improves the texture and feel of products	0.5–2% λ-carrageenan	143 and 144

### 5.1 Dental applications

Sudhakar *et al.* reported that a bio-membrane formulated by combining the carrageenan and polyvinyl alcohol (PVA) along with 3-aminopropyl tri ethoxy silane (APTES) as a cross-linker is stronger than the commercial membrane. The results showed that tensile strength was 89.21 MPa, tensile stress was 3.27%, and contact angle was 78.54° ± 6.11° of bio-membrane. Bio-membrane wettability meets international standards. From all these results, it was concluded that this bio-membrane can be used for dental and other medical applications because of its high mechanical strength and other properties. The bio-membrane having carrageenan is useful for dental tissue regeneration because it initiates the growth of fibroblast cells.^116^

### 5.2 Anti-cancer

**Fig. 13:**
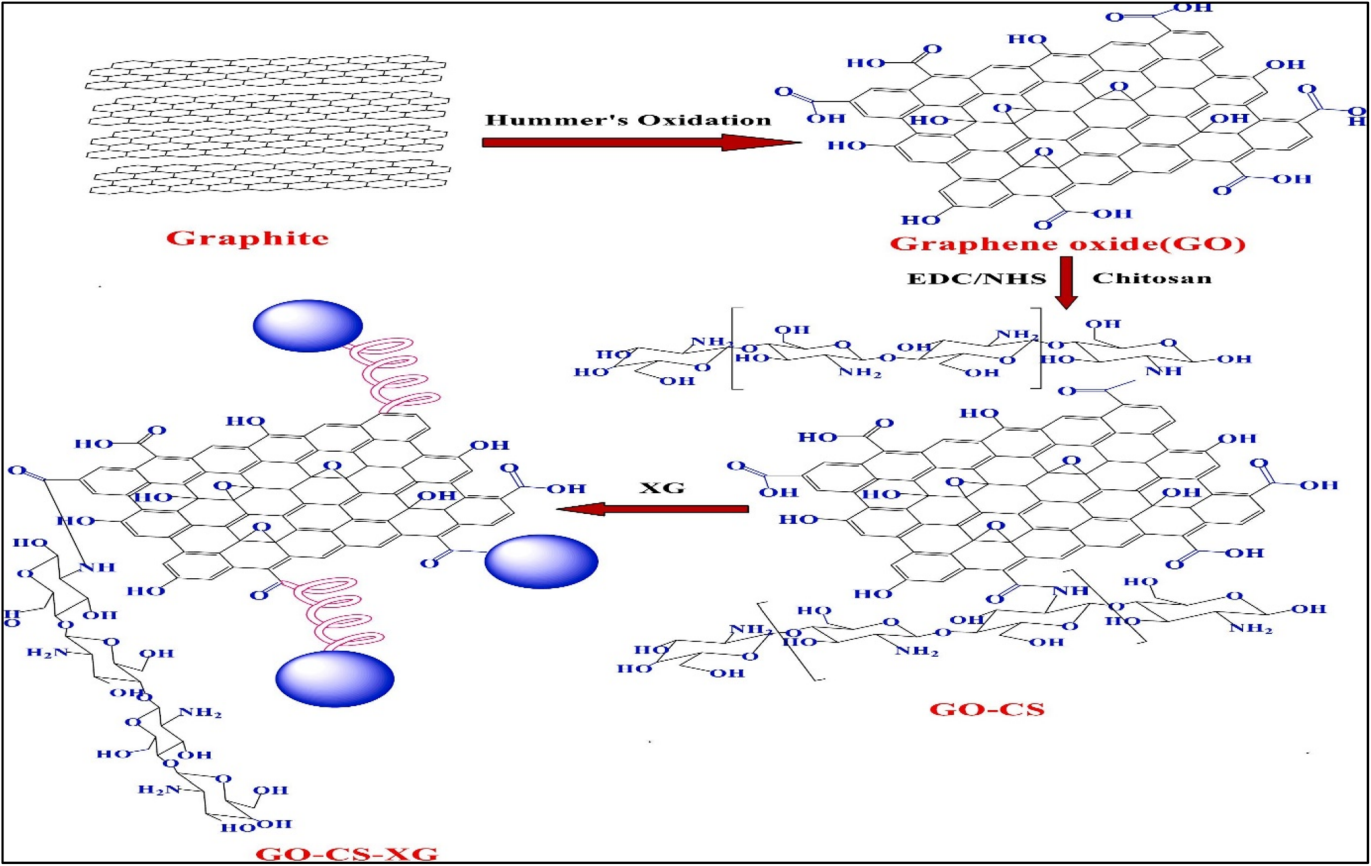
Schematic illustration of GO-CS-XG synthesis. Reprinted with permission from Kesavan S, *et al.*, *International Journal of Biological Macromolecules*, 2023, **31**, 125322. © 2023 Elsevier.^117^

The adaptability of carrageenan as a natural polymer in designing useful nano carriers for cancer therapy has been highlighted by recent developments in drug delivery systems. A notable method uses graphene oxide (GO) modified with chitosan (CS) and capped with xyloglucan (XG) to create a pH-responsive hybrid nano system, and stability is further increased with kappa-carrageenan (Fig. 13). Through validation using FTIR, EDAX, XPS, and HR_TEM studies, this nano-composite showed efficient drug loading (0.422 mg mL^−1^) and regulated release.^117^

In contrast, a comparative study using gallic acid functionalized copper oxide nanoparticles (GaCuO) loaded with paclitaxel (PTX) showed superior ROS-mediated cytotoxicity in MCF _7 cells, with the kappa carrageenan and folic acid coatings enhancing targeted delivery and improved biocompatibility.^118^ Carrageenan-cholesterol (CRG-CHS) and folic acid-modified carrageenan-cholesterol (FA-CRG-CHS) amphiphilic conjugates were synthesized as nano micelle carriers for the intracellular delivery of the anti-cancer drug doxorubicin (DOX). The micelles exhibited excellent drug-loading capacity, sustained drug release in acidic environments, and were non-cytotoxic. The FA-modified micelles showed significantly greater cytotoxicity against folate receptor-overexpressing MDA-MB-231 breast cancer cells compared to MCF-7 cells. These findings indicate that the synthesized CRG-CHS and FA-CRG-CHS micelles are promising carriers for the targeted intracellular delivery of hydrophobic anti-cancer drugs.^119^ Furthermore, carrageenan cholesterol amphiphilic conjugates and it's folic acid modified derivative were designed into nano Micelles for the doxorubicin delivery. These carriers demonstrated acid-responsive sustained release, and FA-targeted micelles showed receptor-mediated selectivity by exhibiting increased cytotoxicity against MDA_MB_231 cells that overexpress folate receptors in comparison to MCF-7^119^ study. In contrast to micelle systems, imatinib mesylate-loaded CS/kC nanoparticles using poly (sarcosine) were utilized in another research on colorectal cancer. This study showed that the nanoparticles had low toxicity and prolonged release over 24 hours at acidic pH, highlighting their potential in colon-specific treatment.^120^ Additionally, in another experimental study, poly(*N*-isopropylacrylamide) (PNIPAm) and carrageenan were combined to create DOX-loaded nanocarriers using gamma irradiation. These nano-carriers showed a theranostic dimension not explored in earlier formulations by effectively suppressing HepG-2 and MCF-7 cells and showed *in vivo* tumour targeting using 99 m Tc- radio labelling. Jafar *et al.* created pH–pH-sensitive k- CG/CS hydrogels with magnetic MMT for sunitinib administration using a green synthesis delivery. Because of electrostatic interactions, this hydrogel system utilized k-CG's intrinsic anionic character at physiological pH, enabling regulated and sustained release profiles.^121,122^ Jafari *et al.* reported the development of pH-responsive and magnetic κ-CG/CS hydrogels incorporating MMT through an entirely green approach for the controlled release of the anticancer drug sunitinib. This was achieved *via* the ionic cross-linking of two natural polymers, κ-CG and CS, in the presence of magnetic MMT (mMMT) nanoplatelets. Due to its low p*K*_a_ value (p*K*_a_ < ∼∼2.5), carrageenan exhibits an anionic nature across a broad range of physiological pH levels, enabling electrostatic interactions with cationic drugs, which facilitates sustained drug release.^123^

Comparatively, nanohybrids based on metals and GO show promising activity in targeting and ROS generation, while carrageenan-based micelles and hydrogels excel in biodegradability, sustainability, and environment responsiveness with capabilities for clinical translation. These findings demonstrate the potential of carrageenan in biocompatibility and functional diversity for the accurate and controlled delivery of anti-cancer agents.

### 5.3 As a macromolecular crowding agent

Macromolecular crowding has emerged as a promising approach to accelerate extracellular matrix (ECM) deposition and the development of functional tissue-engineered constructs. However, the optimal macromolecular crowding agent remains elusive. This study evaluated the biophysical properties and effects of different carrageenan molecules on human umbilical cord-derived mesenchymal stromal cells. The results showed that lambda medium viscosity carrageenan at 10 and 50 μg mL^−1^ concentrations enhanced ECM deposition without affecting cell viability, proliferation, or phenotype. These findings highlight the potential of lambda carrageenan as an effective macromolecular crowding agent for the rapid fabrication of robust cell sheets for regenerative medicine applications.^121^

### 5.4 Drug delivery system

Recent studies have focused on the encapsulation of bioactive compounds, such as curcumin, in polymer-based delivery systems to enhance their stability and bioavailability. κ-Carrageenan hydrogel beads have emerged as promising carriers due to their biocompatibility and ability to form strong hydrogen bonds and ionic interactions with encapsulated substances. The encapsulation efficiency and thermal protection provided by κ-carrageenan enhance the stability of curcumin, facilitating a controlled, pH-dependent release profile. This advancement underscores the potential of κ-carrageenan hydrogel beads in food and pharmaceutical applications for the sustained release of bioactive compounds.^145^ This research demonstrates a green one-pot biosynthesis of silver nanoparticles (SNPs) with varying silver (Ag) ratios in N, N, *N*-trimethyl chitosan chloride (TMC) and carboxymethyl kappa-carrageenan (CMKC). The resulting silver nanocomposites (SNCs) showed enhanced 5-fluorouracil (5-FU) encapsulation, reaching 92.16% with 3% Ag, and achieved sustained 5-FU release up to 96.3% over 24 hours at pH 7.4. Additionally, SNC 3% exhibited strong cytotoxicity against HCT116 cells, good biodegradability, and antimicrobial properties, making it a promising candidate for controlled drug delivery and antibacterial applications.^146^ This study reports the development of pH-responsive magnetic nanocomposite hydrogels for targeted drug delivery in cancer treatment. The nanocomposites, synthesized using Mentha plant extract and a hybrid hydrogel of k-carrageenan and chitosan, demonstrated controlled drug release in a pH-dependent manner, high antibacterial activity, and good biocompatibility, making them a promising carrier for antibacterial and anticancer applications.^147^ This study improved mefenamic acid (MAC) release by crosslinking κ-carrageenan/sericin blends, enhancing entrapment efficiency and drug loading while ensuring gastro-resistance. Characterization confirmed stable drug encapsulation and sustained release, with increased cell viability *in vitro*, suggesting potential for anti-inflammatory therapy with reduced cytotoxicity.^148^ This study reports that carrageenan functions as a biocompatible platform for the delivery of pharmaceutical drugs and facilitates gene delivery.^149^ This study reports that carrageenan-based hydrogels, such as the CAR/DEMA/Gelt/ZnO nanocomposite, are effective for localized drug delivery due to their enhanced antibacterial and anticancer properties.^150^ This research demonstrates numerous carrageenan-based structures, including hydrogels, microparticles, and nanoparticles, which are employed to deliver therapeutic medications and bioactive compounds efficiently. This study also emphasises the difficulties and opportunities associated with the use of carrageenan in medication delivery systems.^151^

### 5.5 Tissue engineering

Advancements in tissue engineering have highlighted the need for biocompatible nanocomposite films with enhanced properties for biomedical applications. Studies have shown that carrageenan can be utilized to develop hydrogels with high molecular weight, suitable for food applications and with potential immune-modulating properties.^152,153^ Furthermore, carrageenan has been evaluated as a macromolecular crowding agent, specifically the lambda medium viscosity type, which has demonstrated the ability to enhance extracellular matrix deposition without compromising cell viability or phenotype, making it a valuable component for tissue engineering applications.^154^ Additionally, carrageenan has been incorporated into composite hydrogels with red blood cell membrane vesicles, showing enhanced mechanical properties and controlled release of hydrophobic drug molecules, highlighting its potential in soft tissue engineering and drug delivery systems.^155,156^ This study successfully fabricated aldehyde-modified carrageenan/gelatin/halloysite nanotube (AD-Carr/Gel/HNTs) nanocomposite films *via* solution casting, incorporating HNTs at varying concentrations. Characterization techniques, including SEM, TGA, mechanical testing, water adsorption, and *in vitro* degradation, confirmed the films' promising attributes. Hemocompatibility and MTT assay results demonstrated that these films are non-toxic and suitable for tissue engineering applications.^157^ Carrageenans are biopolymers derived from red seaweeds, traditionally used in food products as emulsifiers, stabilizers, and thickening agents. They are bioactive polysaccharides with disease-modifying and microbiota-modulating activities, and their biomedical applications include fabricating hydrogels and nanostructures. Recent advances include targeted drug delivery systems and bioink materials for 3D printing in tissue engineering and regenerative medicine.^112^ Another related study developed intricate and interconnected porous mats using natural, bioactive, and biodegradable polymers like polycaprolactone (PCL), chitosan (CS), and κ-carrageenan (κ-C), crosslinked with 1,4-butanediol diglycidyl ether (BDDE). The variation in the formic acid (FA)/acetic acid (AA) solvent ratio significantly affected the fibre mat characteristics, with the polysaccharides and BDDE playing a major role in tailoring the mechanical properties. *In vitro* assessment showed good proliferation of MC3T3-E1 cells on the electrospun fibre mats, indicating their potential for biomedical applications like soft and bone tissue regeneration.^158^ This research showed that sulfated polysaccharides, especially fucoidan and carrageenan, can encourage osteogenic, adipogenic, and chondrogenic development in stem cells. Alginate's ability to create gels strengthens its function as an extracellular matrix that promotes tissue formation and cell proliferation^159^

### 5.6 Wound healing application

**Fig. 14:**
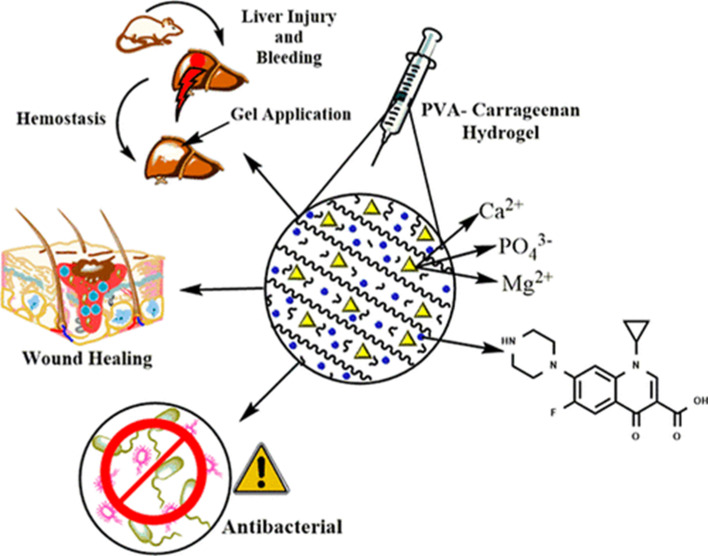
Strategic wound healing application of WNA. Reprinted with permission from Nikhil Kumar, *et al.*, *Biomacromolecules*, 2024, **25**, 1228–1245. © 2024 ACS.^165^

The study successfully developed AgNPs/iota-carrageenan/cotton nanocomposites using ultrasonic waves, leading to increased absorbance values of AgNps at 438 nm and effective reduction of AgNPs with colour hue changes. Incorporation of iota-carrageenan reduced zeta potential values, particle sizes, and enhanced interactions between AgNps and iota-carrageenan. Thermal stability decreased, friction coefficient increased, favouring wound healing, antimicrobial treatment, and drug delivery, with no observed reduction in mechanical properties or cytotoxicity against human skin fibroblast cells.^160^ Studies revealed that κ-Carrageenan (κ-Car) has been used to develop biopolymer composite materials with coriander essential oil (CEO) for wound healing. The CEO-loaded κ-Car films, created through homogenization and ultrasonication, demonstrated effective encapsulation, controlled CEO release, and improved fibroblast adhesion, F-actin organization, and collagen synthesis. These κ-Car-CEO films showed promising *in vitro* and *in vivo* results for promoting wound healing, highlighting their potential in regenerative medicine.^161^ The study focused on creating nanofiber mats using polyvinylpyrrolidone (PVP), PVP/κ-carrageenan (KG), and ursolic acid (UA) for wound dressing applications. The optimized PVP/KG/UA nanofibers exhibited improved properties such as smaller diameter, high porosity, hydrophilicity, degradation rate, wound closure rate, cell proliferation, and antibacterial activity, making them promise for wound healing. Overall, UA-loaded nanofibers showed excellent potential as effective wound dressing materials.^162^ An innovative hybrid super porous hydrogel (MHSPH) combining Dillenia Indica Fruit Mucilage (DIFM), carrageenan, and green-synthesised magnesium oxide nanoparticles (MNPs) was developed for wound dressing. Characterization confirmed its mechanical strength, safety for biological use, and accelerated wound healing in murine models, suggesting its potential as both a dressing material and tissue regrowth scaffold. MHSPH's optimized formulation and MNPs exhibited favourable properties, including biocompatibility, low toxicity, and enhanced wound closure compared to control groups.^163^ Carrageenan, a naturally derived polysaccharide from red seaweeds, is a promising biomaterial for tissue engineering, regenerative medicine, and drug delivery due to its unique properties, like antiviral, immunomodulatory, anticoagulant, antioxidant, and anticancer activities. Chemical modifications enhance its versatility, while research focused on formulating scaffolds and drug delivery systems using carrageenan for tissue repair and disease treatment. Its inherent bioactivity and biocompatibility make carrageenan an appealing candidate for various biomedical applications.^121^ Related studies formulated pH-sensitive hydrogels based on kappa-carrageenan/guar gum/poly (vinyl alcohol) crosslinked with vinyltriethoxy silane, demonstrating controlled drug release and cytocompatibility for potential biomedical applications, including targeted and oral drug delivery. The study investigated physicochemical properties, swelling behaviour, drug release patterns, and cytotoxicity, highlighting the hydrogel's versatility and suitability for various biomedical uses.^164^ Further study developed hybrid nanocomposite cryogels to enhance hemostasis and provide long-lasting antibacterial effects. By cross-linking poly (vinyl alcohol) and κ-carrageenan through freeze-thaw cycles and incorporating whitlockite nanoapatite (WNA) particles and ciprofloxacin, a 3D microporous gel was achieved. The resulting cryogel demonstrated excellent swelling, low cytotoxicity, and strong mechanical properties. With 4% WNA, the gel enabled extended drug release of 71.21% over 21 days, exhibited notable antibacterial activity, and promoted rapid hemostasis in rat models, averaging 83 seconds. Wound healing was accelerated, achieving 96.34% contraction within 14 days, compared to approximately 78% in controls. Histopathology confirmed re-epithelialization by day 14, highlighting this cryogel's promising potential for wound management through effective hemostasis and infection control. The mechanism is shown in Fig. 14.^165^

### 5.7 Blood clotting

Advancements in tissue engineering have highlighted the need for biocompatible nanocomposite films with enhanced properties for biomedical applications. This study successfully fabricated aldehyde-modified carrageenan/gelatin/halloysite nanotube (AD-Carr/Gel/HNTs) nanocomposite films *via* solution casting, incorporating HNTs at varying concentrations. Characterization techniques, including SEM, TGA, mechanical testing, water adsorption, and *in vitro* degradation, confirmed the films' promising attributes. Hemocompatibility and MTT assay results demonstrated that these films are non-toxic and suitable for tissue engineering applications.^166^

**Fig. 15:**
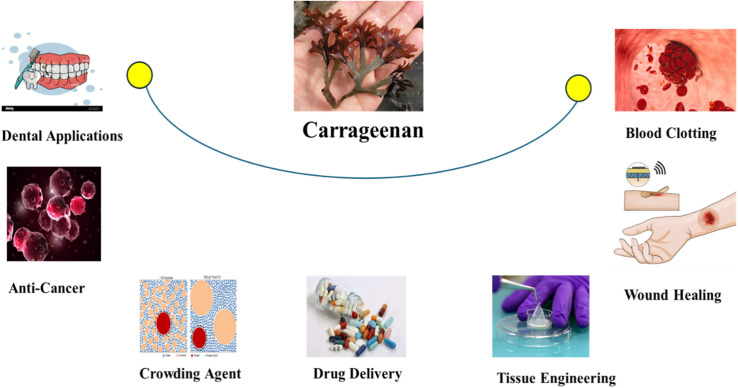
Various applications of carrageenan in biomedical field.

Advancements in tissue engineering have highlighted the need for biocompatible nanocomposite films with enhanced properties for biomedical applications. This study successfully fabricated aldehyde-modified carrageenan/gelatin/halloysite nanotube (AD-Carr/Gel/HNTs) nanocomposite films *via* solution casting, incorporating HNTs at varying concentrations. Characterization techniques, including SEM, TGA, mechanical testing, water adsorption, and *in vitro* degradation, confirmed the films' promising attributes. Hemocompatibility and MTT assay results demonstrated that these films are non-toxic and suitable for tissue engineering applications.^167^ Carrageenan-based hydrogels have been evaluated for their blood compatibility, specifically focusing on thrombogenicity and haemolysis. The results indicate that these hydrogels exhibit non-thrombogenic and non-hemolytic properties, making them suitable for biomedical applications involving blood contact. The study found thrombogenicity levels of 73.40 ± 1.75% and hemolysis at 4.53 ± 0.15%, both within acceptable limits. Carrageenan's anticoagulant properties, like heparin, help prevent platelet aggregation and clot formation, contributing to its potential use in such applications.^168^ Kappa-carrageenan has been successfully incorporated into chitosan-based injectable hydrogels to enhance blood clotting applications. A study found that adding a GO/CaCO_3_/SiO_2_ nanocomposite to carrageenan/chitosan hydrogels significantly improved their hemostatic properties. The sulfate groups in the hydrogel play a key role in activating Factor XII, initiating the intrinsic coagulation pathway. Additionally, calcium carbonate promotes rapid hemostasis by facilitating the conversion of prothrombin to thrombin, accelerating the clotting process. This hydrogel has demonstrated strong potential as an advanced wound care material by effectively reducing blood loss and shortening clotting time in *in vivo* models.^166^ This research demonstrates that kappa-carrageenan, a polysaccharide, is widely used in experimental models to induce thrombosis, enabling the evaluation of anticoagulant therapies. Its role in blood clotting stems from its ability to activate inflammation-mediated coagulation pathways, which are essential for assessing antithrombotic treatments. The endothelial damage caused by κ-carrageenan triggers platelet aggregation and fibrin deposition, making it a valuable model for studying the effectiveness of anticoagulant drugs. Moreover, its structural properties allow for its incorporation into biomaterials designed for hemostatic applications. Its pro-coagulant activity is particularly useful in cardiovascular research, providing a controlled system for investigating thrombosis and potential treatment strategies.^169^ All the applications of carrageenan in the biomedical field are present in Fig. 15.

## 6. Food industry

### 6.1 Food packing

Protein-based films, soy proteins combined with k-carrageenan, enhanced with bacterial cellulose nanofibrils and increased their morphological, water vapor barrier, mechanical and moisture content properties of the films. Zenian loaded metal framework incorporated into the film notably increased its thermal stability, antimicrobial property and antioxidant activity and made it suitable for food packing.^170^ The following studies used the cross linked gelatin films with k-carrageenan and cyclodextrin which shows positive impacts on packing. Cyclodextrin was oxidized with sodium periodate to introduce the aldehydic group allowed for the solubilization of the carvacol which eradicate the rate of antibacterial activity up to 99.98% against listeria monocytogenes and enhance the thermal stability of the film.^171^ In the following studies, the researchers prepared multilayer films with an outer layer of sodium alginate and an inner layer of carrageenan and gelatin containing organo oil and zinc oxide nanoparticles. Different characterization techniques, such as FTIR and SEM, show that multilayer films revealed better UV resistance, higher organo-essential oil and stronger antioxidant and antibacterial properties compared to monolayer films and concluded that multilayer films have greater potential as active food packaging material.^172^ Recent studies on carrageenan-based active and intelligent packaging films show they can extend food shelf life and serve as sensors to monitor spoilage in real-time food packaging tests. Scientists formulated Biodegradable “smart films” made from gelatin, κ-carrageenan, TiO_2_ nanoparticles, and natural pigments (saffron or red barberry anthocyanins) that have the ability to detect food spoilage and extend shelf life. These films change colour in response to ammonia from fish degradation, showing freshness levels. TiO_2_ and anthocyanins improve moisture resistance, antimicrobial activity, and block light, enhancing food quality. The films are eco-friendly, decomposing in about 30 days, and offer a sustainable alternative to plastic packaging.^173^ This study reports the use of carrageenan in food preservation and in biodegradable food packaging by serving as a barrier against moisture loss and microbiological contamination. Carrageenan-based films have potent antioxidants and antibacterial qualities that can prolong the shelf life of perishable foods when paired with bioactive substances like plant extracts. By reducing oxidation and microbial growth, these coatings are especially helpful for meat and seafood, maintaining texture and freshness. Furthermore, carrageenan-based microcapsules enhance the stability and safety of food.^174^ This study explored carrageenan-based TiO_2_ composites with antibacterial properties against *S. aureus* and *E. coli*, enhancing food preservation, extending shelf life, and improving biodegradability in packaging. Their combination improves stability, mechanical properties, and antimicrobial effectiveness, offering a sustainable alternative to synthetic preservatives.^175^Table 5 shows the application of carrageenan in the food industry and its potential application areas, with the mechanism of action.

**Table 5:** Application of carrageenan in food industry and its potential application areas with mechanism of action

Application area	Description	Mechanism	Advantages	Preferred amount/Type	Ref.
Thickening agent	Carrageenan is widely used to enhance the viscosity of food products	Forms a gel-like structure in solution, increasing thickness	Improves texture and mouthfeel; enhances product stability	0.5–1.5% κ-carrageenan	176
Stabilizer	Helps maintain the uniform distribution of ingredients in food products	Prevents separation by stabilizing emulsions and suspensions	Extends shelf life; maintains product quality	0.2–1% λ-carrageenan	177
Gelling agent	Provides gelation properties in various food applications	Forms gels upon cooling or upon interaction with other ingredients	Versatile for desserts, dairy, and meat products	1–2% ι-carrageenan	178
Emulsifier	Used to stabilize oil-water mixtures in food products	Reduces surface tension between immiscible liquids	Enhances texture; improves flavour release	0.5–1% κ-carrageenan	179
Fat replacement	Carrageenan can mimic the texture of fat in low-fat and reduced-calorie foods	Provides creaminess and mouthfeel without added calories	Supports healthier formulations, appealing to health-conscious consumers	0.5–1.5% λ-carrageenan	180
Clarifying agent	Used in beverages to remove cloudiness and improve appearance	Binds with suspended particles, aiding in their removal	Enhances visual appeal; improves consumer perception	0.1–0.5% κ-carrageenan	176
Coating agent	Carrageenan can be used as a coating for food products to retain moisture	Forms a barrier that reduces moisture loss and enhances shelf life	Extends freshness; improves texture	1–2% ι-carrageenan	181
Plant-based alternatives	Utilized in vegan and vegetarian products to replicate texture and mouthfeel	Mimics the properties of animal-derived gelling agents	Supports the growing demand for plant-based foods	0.5–2% blended carrageenan	182
Beverage stabilization	Helps stabilize fruit juices and dairy beverages, preventing sedimentation	Maintains homogeneity by preventing the separation of phases	Improves product consistency; enhances consumer experience	0.5–1% λ-carrageenan	183
Dessert products	Commonly used in puddings, mousses, and jellies for texture and stability	Forms a gel that provides structure and creaminess	Versatile in various dessert applications; enhances indulgence	1–2% κ-carrageenan	184

### 6.2 Shelf-life extension

The following study reveals that the addition of carrageenan to meat sausages increases their properties like microbiological, texture, moisture, emulsion stability and cohesiveness. Carrageenan addition reduces fat, increases firmness and extends shelf life. Sensory analysis shows that carrageenan has no effect on sausages taste and maintains the desired product quality.^185^

**Fig. 16:**
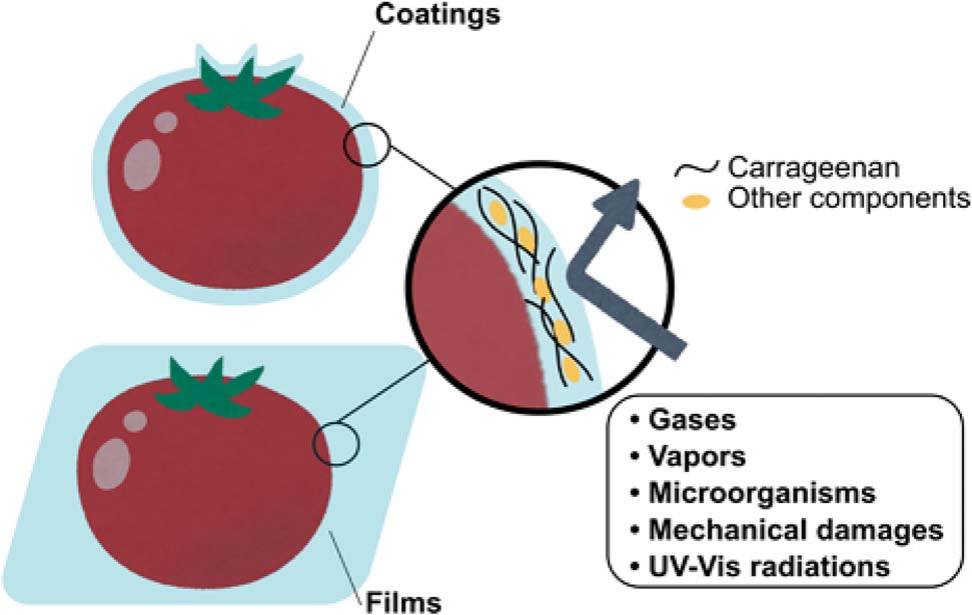
Shelf-life extension of cherry tomatoes by carrageenan coating. Reprinted with permission from Toshifumi Udo *et al.*, *Food Research International*, 2023, **173**, 113369. © 2023 Elsevier.^15^

Another study shows the advantages of carrageenan when mixed in flour dough for baked products. kc-carrageenan is very popular and increases the water retention properties, resulting in a moist and soft texture, extending shelf life and preventing dryness.^186^ Carrageenan film can also aid in vegetable storage. Studies showed that tomatoes coated with a biodegradable edible film made from arrowroot starch and ι-carrageenan remain firmer compared to unwrapped tomatoes.^187^ Another research study revealed that Konjac glucomannan-SiO_2_-carrageenan composite nanofilms can minimise weight loss and oxygen permeability in white mushrooms, which slows their respiration rate and reduces browning. This extends the shelf life of mushrooms stored at 4 °C by an additional 5–12 days.^188^ Refrigeration and freezing are widely used to preserve fish and meat products, but frozen versions often lack the desired flavour, making refrigerated options more popular. To prolong the shelf life of these cold products, there is growing interest in using carrageenan-based composite films. For instance, researchers performed an experiment and revealed that coating beef with copper sulphide nanoparticle-carrageenan films has been found to lower *Escherichia coli* and *Staphylococcus aureus* counts by 52.6% and 69.8%, respectively, compared to uncoated beef.^189^ This study evaluates the use of oregano essential oil encapsulated in gelatin microcapsules with carrageenan for food preservation. The encapsulation achieved an efficiency of 87.79% and sustained release for over 80 hours, effectively inhibiting *Botrytis cinerea*. This approach extended the shelf life of cherry tomatoes by reducing fungal infection and maintaining their quality, demonstrating the potential of oregano essential oil as a natural food preservative.^190^ This study demonstrates carrageenan-based antimicrobial hydrogel coatings for food preservation by reducing microbial contamination. K-carrageenan infused with carvacrol/hydroxypropyl-β-cyclodextrin delays spoilage and extends the shelf life of strawberry by reducing weight loss, texture degradation and acidity changes. Its antimicrobial properties inhibits *S. aureus* up to 15 days.^191^ Carrageenan-based edible films, combined with gelatin and bioactive extracts acting as moisture and oxygen barriers. They prevent oxidation and microbial contamination, improving product stability enhance food preservation in cherry tomatoes.^192^Fig. 16 shows the use of carrageenan in packaging material prepared by casting in a mold in advance.^15^

### 6.3 Food preservation

Another researcher shows that K-carrageenan in which benzyl iso thiocyanate encapsulated in β-cyclodextrin was used to create the antibacterial food packing film. The prepared film enhance the hydrogel network structure and modulus structure and show antibacterial activity against the *Staphylococcus aureus* and Listeria monocytogenes on chicken meat at 25 degree centigrade and decrease microbial growth in refrigerator at 4 degree centigrade for 8 days indicating its potential for food preservation.^193^ Likewise, carrageenan and camellia oil films used in chicken preservation significantly decreased cryophilic bacteria levels to 3.86 log CFU g^−1^, compared to 5.35 log CFU g^−1^ in the untreated control group.^194^ This study investigated carrageenan-based coatings for food preservation, particularly in meat storage. The incorporation of curcumin-loaded zein nanoparticles enhanced the coating's durability and adhesion, ensuring prolonged freshness. The formulation exhibited strong antioxidant activity (89.78% DPPH• scavenging) and effective antibacterial properties, significantly reducing spoilage microorganisms such as *Pseudomonas fragi* and *Brochothrix thermosphacta*. By minimizing oxidative degradation and microbial growth, the coating extended the refrigerated shelf life of lamb and pork by 1.8 to 2.3 times.^195^ Carrageenan-based films have been developed as a sustainable alternative to synthetic packaging for preserving olive oil. Functionalization with curcumin-loaded solid dispersions improved their water resistance, tensile strength, and barrier properties. Compared to unprotected samples with a peroxide value of 20 mEq O_2_ kg^−1^, these films effectively reduced lipid oxidation, maintaining a lower value of 14 mEq O_2_ kg^−1^. Additionally, they exhibited strong antibacterial activity, reducing *Listeria monocytogenes* by 90.6% and *Escherichia coli* by 95.4%, demonstrating their potential for food preservation and extended shelf life.^196^ Bio nanocomposite films carrageenan based with 20 wt% Ag-doped CeO_2_ demonstrated better antibacterial action than amoxicillin. Additionally, they improved Young's modulus (15.96 GPa) and tensile strength (5.10 MPa) for increased durability. These films are perfect for sustainable food packaging because of the enhanced water resistance provided by Zn-doped CeO_2_ NPs.^197^

### 6.4 Food additives

This study investigates essential and toxic elements in carrageenan and gums that were used as food additives. The researchers found essential elements like Fe, Mn, and Zn in carrageenan. The results showed that daily consumption of food additives at an acceptable level can contribute to the daily requirement of some elements.^198^

This study focuses on carrageenan, a food additive derived from algae and shows that antinutritional effects are nullified in the meat ball matrix and inhibit the proteolysis of soluble collagen it does not interfere with the digestion of meat proteins, suggesting that the matrix effect of meat protects against CGN's potential adverse effects. These are efficient for evaluating the safety and regulatory status of CGN in food products.^199^ This research demonstrates that sulphated polysaccharide-based carrageenan is used as a food additive and is essential for enhancing food texture. It is frequently added to processed meats, dairy products and dishes made with gelatine in amounts between 0.01% and 0.5%.^200^ Carrageenan increases moisture retention, texture and stability, making it essential in dairy and processed foods. When blended with xanthan gum, it enhances elasticity, making it perfect for vegan gummies. In beverages and plant-based foods, their rheological properties aid gel formation, emulsion stabilization, and natural colourant retention, preventing anthocyanin degradation.^201^

### 6.5 Baking industry

In the following study, the researchers used k-carrageenan in cakes that inhibited the formation of end products (AGEs) formed in heat-processed foods and lowered the health risk of diabetes related complications. Analysis of cake characteristics reveals that the cake with 1% w/w k-carrageenan had the highest quality and overall acceptance.^202^ This study explored an ovalbumin–ferulic acid κ-carrageenan Pickering emulsion (OE) as a full butter substitute in bread, focusing on κ-carrageenan's role in enhancing bread structure and quality. Bread with OE showed increased volume, a well-developed gluten network, and improved texture over storage, with high consumer acceptance. κ-Carrageenan contributed to a stable emulsion that created a compact gluten network, effectively embedding starch and maintaining softness, making this emulsion a promising industrial butter alternative for bread.^193^ This study investigated the effects of varying κ-carrageenan (κ-C) levels on the rheological behaviour of a cake flour model system. Higher κ-C concentrations led to increased viscosity and moduli (*G*′ and *G*′′), indicating a firmer texture due to κ-C's entanglement with starch and gluten. Increased κ-C also reduced creep compliance, showing enhanced resistance to deformation, particularly affecting gluten in the system. Additionally, 10 g/100 g κ-C raised starch gelatinization onset temperatures, likely due to its high-water retention, which lowered water activity. These findings highlight κ-C's potential in bakery applications, especially in formulations requiring added structure and stability^203^

### 6.6 Dairy products

**Fig. 17:**
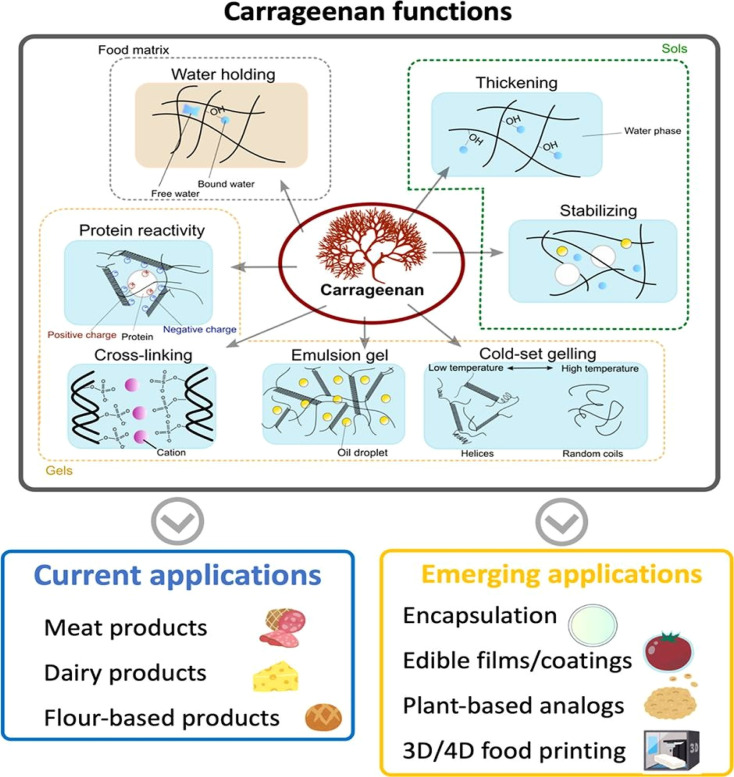
Distinctive characteristics of carrageen leading towards a variety of applications in the food industry. Reprinted with permission from Toshifumi Udo *et al.*, *Food Research International*, 2023, **173**, 113369. © 2023 Elsevier.^15^

Kappa carrageenan exhibits a strong synergy with milk proteins, especially casein, leading to the formation of a milk gel. At low concentrations (100–400 ppm) and in the presence of calcium or potassium ions, a weak gel network forms, which serves as a stabilizer and suspension agent. The interaction between kappa carrageenan and casein strengthens this network in milk, reducing the amount of carrageenan needed to about one-fifth of that required to create a comparable gel in water.^176^ Kappa carrageenan serves as a highly efficient stabilizer and suspension agent in dairy products, such as chocolate milk, due to its strong protein interaction and minimal dosage needs, making it economical. In comparison, iota carrageenan provides moderate protein interaction, while lambda has a low level. Lower reactivity adds viscosity instead of a gelled consistency, which is ideal for creating a creamy texture in dairy desserts. For lambda carrageenan, the quantity required to achieve viscosity in milk is about one-tenth of what is needed in water.^204^ This study developed and optimized an Aloe vera-based edible film, enhanced with carrageenan, to improve microbial and lipid oxidative stability in frozen dairy products. Kulfi was used as a model to test various levels of carrageenan (1.0, 1.5, and 2.0%), glycerol, and Aloe vera extract. The ideal formulation—1.5% carrageenan with Aloe vera—showed strong antioxidant and antimicrobial effects, reducing microbial growth, free fatty acids, and oxidation in kulfi during six months of storage. This carrageenan-based film offers potential as a protective, stability-enhancing layer for frozen dairy items.^205^ Physicochemical properties of carrageenan are increased through acetylation, esterification, ion-exchange, and carboxymethylation, improving stability, strength, and solubility. These modifications make it appropriate for a variety of uses such as a stabilizer, thickener and gelling agent in dairy products, processed meats, and confections, enhancing texture, moisture retention, and shelf life.^149^Fig. 17 shows the different applications of carrageenan as an ingredient in food applications.^15^

## 7. Future directions and bottlenecks of carrageenan-based studies

Despite being promising material for wastewater treatment, biomedical applications and the food industry, carrageenan faces a persistent bottleneck that must be addressed and explored for broader clinical and industrial adoption. One of the main drawbacks in wastewater treatment is the limitation in reusability and regeneration of the carrageenan adsorbents, as structural degradation occurs after repeated cycles. Many of these studies are based on model systems and the absence of data on real industrial effluents containing complex mixtures of pollutants, leading to a question mark on the applicability of adsorbents in large water bodies. This feature is the biggest drawback of small-scale laboratory findings. Large-scale synthesis of material is also another hurdle as the economic viability of these synthetic processes is still out of reach. Keeping these points in mind, future research should prioritize the synthesis of hybrid composites incorporating components like covalent organic frameworks and magnetic nanoparticles to enhance regeneration capacity over multiple cycles as well as stability to ensure maximum results.

In the food sector, carrageenan films employed in food packaging have limitations due to poor water barrier features, which cause spoilage. Most of the approaches currently employed are based on plant extracts that raise the production costs, which complicates the scalability factor. Lack of continuous standardised production methods hinders the industrial adoption of lab methods. Most of these strategies are based on the solvent casting technique, which is not easy to handle and is not trustworthy on a commercial scale. Future studies should focus on low cost and waste derives options to synthesize multifunctional composites and improve barrier properties. Extrusion and 3D printing can be a good, large scale processing technique to enhance and standardize film quality and large-scale productions. Integrating pH-responsive dyes as a smart indicator can add value by real-time monitoring of food freshness.

Carrageenan is a promising biomedical material but faces translational gaps. Most of the hydrogel-based drug delivery systems demonstrate desirable results *in vitro* but lack *in vivo* environments. The relationship between therapeutic outcomes and chemical modifications remains poorly understood. Long-term biocompatibility and toxicity data for certain applications (sensitive tissues such as cartilage) are still very limited. To cope with this issue, future explorations should focus on developing effective stimulus-responsive systems for targeted drug delivery, mainly in cancer therapy. Designs and optimization of hydrogels can be done through machine learning and computational modelling. *In vivo* studies should gather long-term data to ensure safety.

Another important hurdle in standardization and reproducibility of these materials in every application is the feedstock variability due to varying seaweed sources. Despite being advertised as a sustainable material, the effects of chemical modifications are not much studied. There is still a lack of regulatory frameworks, standardized protocols and safety assessments for the use of carrageenan-based products in the pharmaceutical and food sector. Interdisciplinary efforts are needed at the time to fully use the potential of carrageenan. Utilizing waste-to-resource pipelines (algal biorefineries) can reduce cost and sustainability problems. Collaborations across industrial engineering, materials science and computational modelling are needed to translate laboratory methods into commercial applications.

**Fig. 18:**
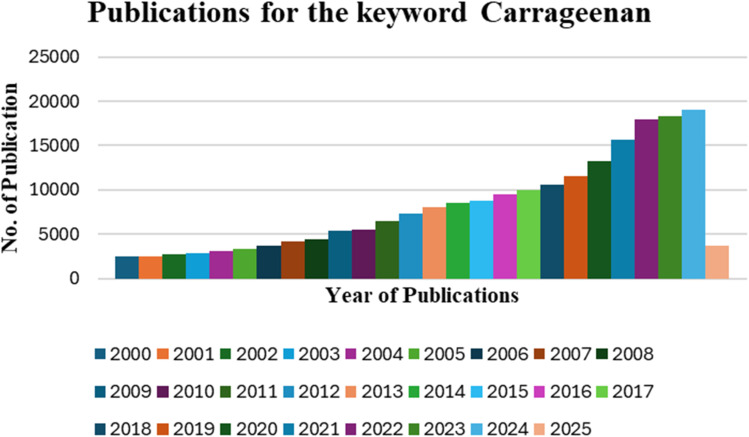
Publications for the keyword carrageenan on science direct.

Fig. 18 shows trends and publications in carrageenan-based studies. Data was collected through ScienceDirect's database, which compiles annual publication counts from the year 2000 through 2025. It is expected that the tendency will continue in 2025.

## 8. Conclusion

This review highlights carrageenan's significant potential as a versatile biomaterial with applications across environmental remediation, biomedical sciences, and food technology. In wastewater treatment, carrageenan-based materials have demonstrated remarkable efficacy in pollutant removal, while in biomedical fields, they show promise for drug delivery systems and tissue engineering. Carrageenan's continued importance in the food industry is evident, with emerging applications in active packaging. However, challenges remain, including concerns about gastrointestinal effects and the environmental impact of seaweed harvesting. Future research should focus on optimizing carrageenan modification techniques, exploring novel applications, and developing sustainable production methods. As investigations continue to reveal new aspects of this polysaccharide, carrageenan is positioned to play a crucial role in addressing global challenges related to environmental protection, healthcare advancement, and food security. Its unique properties at the intersection of sustainability and innovation make carrageenan a key contributor to the development of eco-friendly solutions across multiple sectors, potentially driving significant advancements in sustainable technologies and biomedical applications.

## Conflicts of interest

The authors declare no competing interests relevant to this study.

## Data Availability

The data supporting the findings of this study are available from the corresponding author upon reasonable request.
